# Early Cardiac Intervention Decreases the Mortality Risk Linked With Marfan Syndrome

**DOI:** 10.7759/cureus.82910

**Published:** 2025-04-24

**Authors:** Suleman Janjua, Andrew Lew, Sergio Sokol, Parto Jalali, Unique Andres, Diane Olski

**Affiliations:** 1 Sports Medicine, Ascension Providence Hospital, Southfield, USA; 2 Sports Medicine, William Carey University College of Osteopathic Medicine, Hattiesburg, USA; 3 Cardiology, Mount Sinai Hospital, New York, USA; 4 Family Medicine, UT Health San Antonio, San Antonio, USA; 5 Emergency Medicine, University of Iowa Health Care, Iowa City, USA; 6 Family Medicine, Grand Strand Medical Center, Myrtle Beach, USA

**Keywords:** aortic root dilation, connective tissue disorder, heart failure with reduced ejection fraction, marfan syndrome, mitral valve prolapse

## Abstract

Early cardiovascular intervention, such as valve repairs, can extend the life expectancy of patients with Marfan syndrome (MFS) by improving blood flow, preventing regurgitation, and optimizing preload and afterload. These improvements enhance ventricular function, ultimately reducing both cardiac and respiratory symptoms. More commonly, patients with MFS can develop aortic root dilation and mitral valve prolapse as a result of a diseased valve. The combination of cardiac repairs and proper medication adherence can decrease the mortality risk linked with MFS. More specifically, aortic and mitral valve repairs can restore the functions of the aorta, left ventricle, and left atrium, respectively, and reduce further complications leading to aortic dissection and heart failure.

## Introduction

Marfan syndrome (MFS) is an autosomal dominant connective tissue disorder affecting males and females equally, most commonly in patients with a positive family history. MFS is expected in affected families, although up to 25% of patients can have a de novo mutation resulting in MFS. The defective gene in MFS is the fibrillin-1 gene (FBN1), which encodes for the connective tissue protein fibrillin-1. Current evidence also suggests that this gene influences the activity of growth factors, such as transforming growth factor beta (TGF-β). The dysregulation of TGF-β contributes to many of the disease’s pathological features, including aortic aneurysm formation. This mutation can range in phenotypic presentations, with variation from mild skeletal features to full systemic involvement, most showing at least some overlap with the classic Marfanoid presentation. MFS is the most inherited connective tissue disease; however, the incidence is 1 in 3,000-5,000 individuals, with less than 200,000 US cases per year [1]. Due to the low disease incidence, a patient with Marfan can be a considerably rare find.

MFS covers an extensive range of symptom presentation and severity depending on organ involvement and phenotypic expression of a mutation. A typical isolated feature seen often involves the aorta, as approximately 60-80% of patients will present with aortic root dilatation and subsequent aortic regurgitation on ECG, although it may not always be present. In some cases, the regurgitation is a result of progressive dilation. These cardiovascular symptoms are the main cause of morbidity and mortality in these patients. Other pathognomonic features are found within the ocular and musculoskeletal systems. Some examples are arachnodactyly, pectus carinatum, scoliosis, ectopia lentis, and retinal tears/detachments. These features are included in a systemic score criterion, which helps to stratify the severity of connective tissue involvement. Although the cardiovascular, ocular, and musculoskeletal systems are the most commonly affected, it has also been known to affect the pulmonary, integumentary, and central nervous systems [1].

Diagnosis is made by the revised criteria, Ghent nosology, which considers a family history of aortic root dilatation, patient history of dilatation, or an FBN1 mutation that was previously associated with aortic root dilatation. This criterion emphasizes the strong predisposition for vascular disease [2]. In ambiguous cases, particularly to distinguish MFS from other heritable aortopathies, genetic testing can help to make the diagnosis. MFS differs from other connective tissue disorders, such as Loeys-Dietz syndrome (LDS) or Ehlers-Danlos syndrome (EDS), in both its genetic mutations and pattern of organ involvement. For instance, LDS often presents with more aggressive vascular pathology and craniofacial abnormalities, while vascular EDS is characterized by fragile blood vessels and skin hyperextensibility due to COL3A1 mutations. Distinguishing between these syndromes is crucial for prognostication and management. Complications of MFS often stem from the patient either being undiagnosed or untreated. The most frequent complication found is aortic dissection, typically a type A in the Dailey scheme or a type I dissection in the DeBakey classification [3]. This is often described as the dissection beginning just above the coronary ostia and extending the length of the aorta [1]. Prophylactic aortic root surgery has dramatically improved survival rates, with elective repair reducing the risk of dissection compared to emergent surgery [3,4].

Understanding the natural history of MFS is essential for improving outcomes. Without intervention, progressive aortic dilation often culminates in type A aortic dissection, the most common and life-threatening complication [3]. Historically, many patients with MFS did not survive past early adulthood. However, prophylactic aortic root replacement and modern cardiovascular surveillance have dramatically improved life expectancy, sometimes approaching normal lifespan when diagnosed and managed early. Delayed diagnosis, on the other hand, may lead to missed opportunities for timely surgical intervention or medical therapy, increasing the risk of irreversible cardiomyopathy, arrhythmias, or sudden cardiac death.

## Case presentation

This patient is a 47-year-old African American male with an extensive medical history significant for rheumatic heart disease, hypertension, hyperlipidemia, congestive heart failure with an ejection fraction (EF) of 5%, peripheral vascular disease, presbyopia, and posterior vitreous detachment, who was recently diagnosed with MFS late in life. Diagnosis was primarily clinical, based on revised Ghent nosology with findings such as posterior vitreous detachment, eye floaters, photopsia, prior aortic and mitral valve replacements, and dilated cardiomyopathy. Per patient history, his father had been diagnosed with MFS at the age of 92, but the course of his disease was unknown due to advanced age.

**Figure 1:**
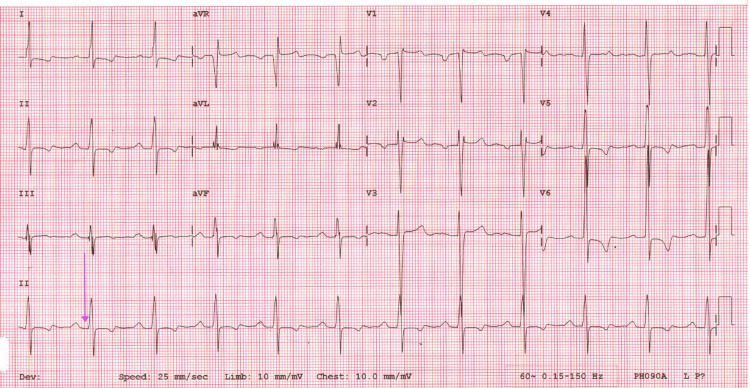
Initial ECG from 2015 showing first-degree AVB, indicated by PR interval prolongation (purple arrow) AVB, atrioventricular block

**Figure 2:**
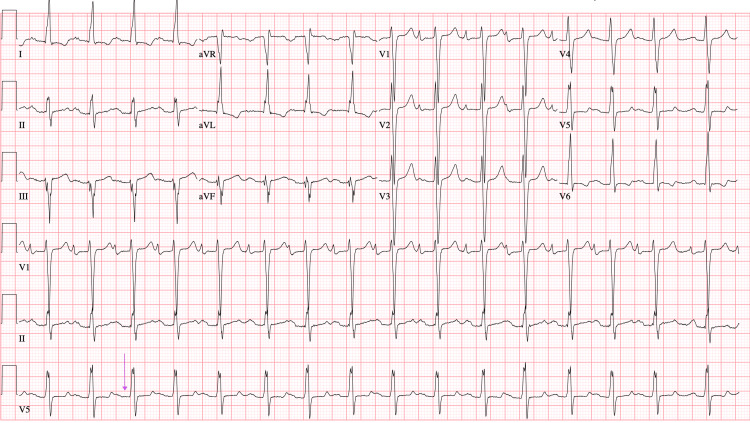
ECG from August 2021 showing first-degree AVB (purple arrow) AVB, atrioventricular block

**Figure 3:**
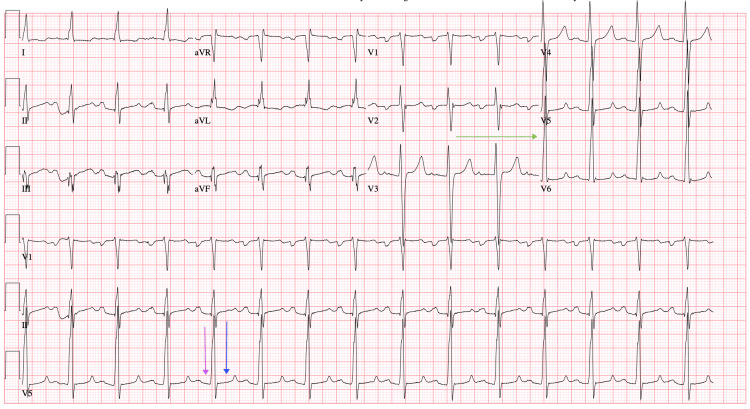
ECG from November 2021 showing first-degree AVB (purple arrow), QT prolongation (blue arrow), and LVH (green arrow) AVB, atrioventricular block; LVH, left ventricular hypertrophy

**Figure 4:**
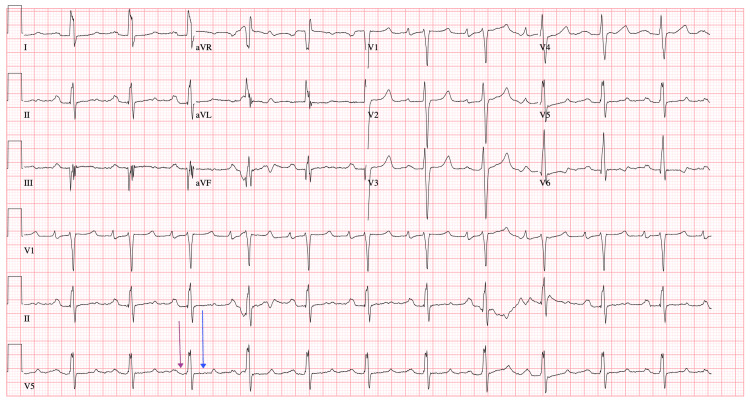
ECG from early January 2022 showing first-degree AVB (purple arrow) and QT prolongation (blue arrow) AVB, atrioventricular block

**Figure 5:**
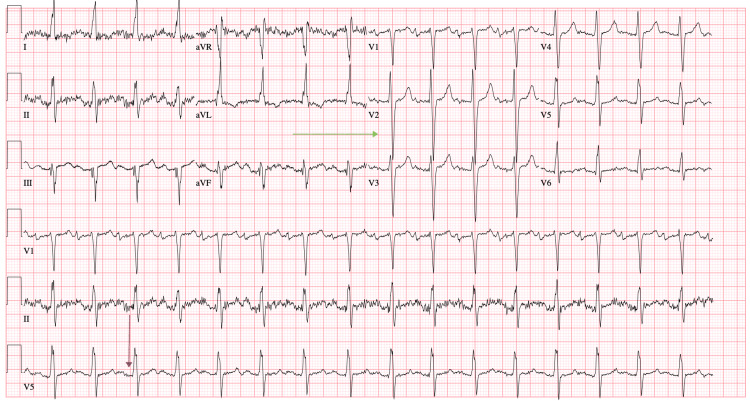
ECG from late January 2022 showing first-degree AVB (purple arrow) and LVH (green arrow) AVB, atrioventricular block; LVH, left ventricular hypertrophy

Six years before the patient’s diagnosis of MFS, his cardiac symptoms were managed with mechanical aortic valve and mitral valve replacement and prescribed lifelong warfarin, but despite intervention, his EF remained low. Initially, the low cardiac output was thought to be related to postoperative changes. Moreover, it was unclear whether the valvular disease had the greatest contribution from the childhood rheumatic fever or the MFS-related pathology. The patient made frequent hospital visits primarily due to exertional chest pain, fatigue, and medication refill needs, often prompting workups for acute coronary syndrome (ACS) or heart failure exacerbations. While chest pain and subtherapeutic international normalized ratio (INR) raised concern for ischemia, serial cardiac enzymes were mildly elevated but generally negative. There was no evidence of obstructive coronary artery disease on previous imaging. During each visit, he was evaluated and admitted for the workup of ACS and myocardial infarctions, for which primary pharmaceutical intervention provided acute symptomatic relief. The inpatient primary and cardiology care teams obtained repetitive ECGs throughout his documented visits that reported a first-degree atrioventricular block (AVB), and ECGs were indicative of dilated cardiomyopathy, cardiomegaly, and heart failure with a reduced EF of 10%, which were consistent with each admission and disposition (Figure 1, Figure 2, Figure 3, Figure 4, Figure 5). The first-degree AVB present on multiple ECGs may more likely be attributed to chronic conduction system disease, possibly from the history of rheumatic fever. The ECG findings were not interpreted as directly related to MFS but were reflective of the patient’s complex cardiovascular comorbidities.

One year before the diagnosis of Marfan in 2021, he was admitted due to another episode of atypical chest pain and subtherapeutic INR, where he developed eye floaters. This prompted an ophthalmology consultation for the diagnosis of photopsia, which determined a history of posterior vitreous detachment at that time. Further, the subtherapeutic INR prompted cardiology consultation and reinforcement of medication compliance. Progressive cardiac symptoms after valvular surgery and ophthalmic involvement raised concern for a concurrent or previously unrecognized connective tissue disorder contributing to the cardiovascular issues that may not be just due to a history of rheumatic fever, prompting further workup that contributed to the Marfan diagnosis despite the absence of obvious skeletal abnormalities.

**Figure 6:**
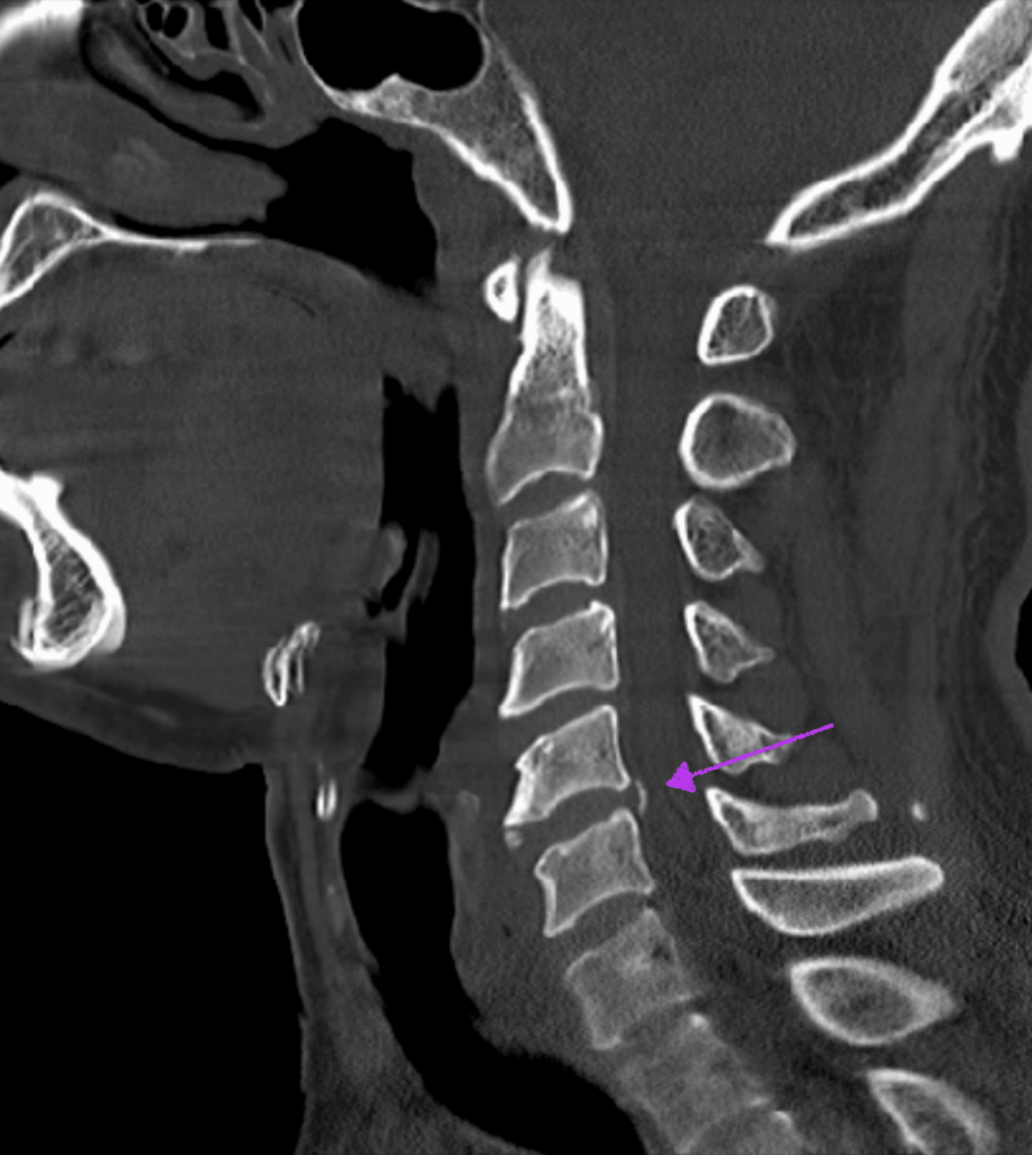
CT showing incidental calcification of the posterior longitudinal ligament (purple arrow) There is no evidence of dural ectasia, which is a more classic spinal manifestation of MFS. MFS, Marfan syndrome

The patient presented to the hospital, yet again, with the complaint of upper extremity paresthesia after undergoing open heart surgery. On this visit, a CT scan of the cervical spine was ordered, which suggested disc protrusions at multiple sites as well as posterior longitudinal ligament calcification (Figure 6). Meanwhile, medication reconciliations were documented with each visit, and the importance of medication compliance was reiterated. The patient’s medication regimen included a potassium-sparing diuretic, loop diuretic, beta blocker, and a fixed-dose combination neprilysin inhibitor with an angiotensin receptor blocker. With all considerations, the patient was a candidate for automatic implantable cardioverter-defibrillator (AICD) placement and underwent this procedure, given the evidence of deterioration with an EF of 5%.

## Discussion

The rarity of MFS makes prompt diagnosis critical to improved prognosis. The sooner the diagnosis is detected, the sooner a regimen for preventative care and regular monitoring can be instilled [3]. Early detection allows for aortic root surveillance, prophylactic intervention, and lifestyle counseling to reduce life-threatening complications. For this patient, the later disease detection raised concerns about rapid cardiovascular deterioration despite valvular and pharmaceutical interventions. Because of these concerns, an AICD placement was suggested. At the time of presentation, the patient had an EF of 5%, which improved modestly to 15% after guideline-directed medical treatment (GDMT) optimization and valve replacement, although he remained at high risk for arrhythmia.

Although the patient’s valvular disease was initially attributed to rheumatic fever, Marfan-associated aortic root involvement was not considered until much later, potentially missing opportunities for earlier surgical planning, such as prophylactic aortic root intervention. The patient did not present with the ophthalmic signs until later, prompting the suspicion of MFS. In this case, intrinsic myocardial dysfunction could not be ruled out, especially given the persistence of severe systolic dysfunction post-valve replacement.

Aortic root dissection and rupture are the leading causes of death in patients with MFS [4]. The frequency of aortic dissection in patients of older age is 2% when compared to 50% in patients under the age of 40 [3]. In this case, the patient had a history of annuloaortic ectasia with a documented ascending aortic diameter of 5.2 cm, contributing to severe aortic regurgitation and mitral regurgitation. Annuloaortic ectasia is a form of aortic root dilation that results in the dilation of the ascending aorta, which more specifically leads to aortic and mitral valve regurgitation, warranting the need for valvular repair and replacement to improve mortality [4].

The concept of “Marfan cardiomyopathy” remains under debate. Primary cardiomyopathy in MFS may stem from intrinsic myocardial dysfunction associated with FBN1 mutations and abnormal TGF-β signaling. Secondary cardiomyopathy often results from volume overload due to aortic or mitral regurgitation, arrhythmias, or intraoperative ischemia. A study from Berlin, published in 2015, took a closer look at patients who were genetically diagnosed with MFS from 1986 to 2013 and who had undergone valvular surgeries and replacement of the aortic root, ascending aorta, and proximal aortic arch. The study aimed to evaluate primary and secondary cardiomyopathy in patients who underwent surgical management for cardiovascular sequelae of MFS. As a result, the study found that secondary cardiomyopathy was attributed to the inherent susceptibility of myocardial ischemia during surgery [5,6].

Utilizing Ghent criteria, there is a priority of cardiovascular features over skeletal and ocular manifestations when family history is absent. Individuals with MFS tend to have excess linear growth of long bones, genetically making them taller than the general population. Pectus deformity, such as pectus carinatum, which gives the chest wall a bulging appearance, can also be examined in such patients, as it is more common in MFS than any other chest irregularity. The appearance of slender, elongated phalanges known as arachnodactyly is also an observed physical trait that this patient also portrayed [1,3,4,7]. A total of 50-80% of patients with MFS develop ectopia lentis, which is an upward lens displacement diagnosed on slit lamp examination [1,3]. The finding of myopia greater than 3 diopters contributes to a feature of this disease [1]. The presence of either scoliosis or kyphosis, as a result of spinal deformities in MFS, can be observed in some patients. In this patient, a combination of systemic features - positive wrist and thumb signs, myopia >3 diopters, mild-moderate scoliosis confirmed on imaging, and a history of family members with aortic disease - supported a clinical diagnosis of MFS. Genetic testing is useful in borderline cases, especially when differentiating from other heritable aortic diseases like LDS. In this patient, genetic testing was not performed as Ghent systemic score criteria were met. His PR interval was also mildly prolonged, although not diagnostic, and no delta wave was seen. Despite the various presentations, the Ghent diagnostic criteria of MFS rely more on the presence or absence of family history, aortic root dilation, FBN1 gene mutation, and the specific findings of ectopia lentis [2,3]. In summary, this case illustrates the therapeutic complexity of managing advanced heart failure in a patient with late-diagnosed MFS. While valve replacement and GDMT improved symptoms modestly, persistent low EF necessitated device therapy. Improved recognition of systemic signs and early referral for genetic testing or cardiology evaluation may help prevent similar progression in future cases.

## Conclusions

It appears that MFS, in its rarity, can benefit from intervention at any stage, although earlier detection offers the best opportunity to prevent life-threatening complications. As depicted in the case of this 47-year-old male, late diagnosis resulted in advanced cardiovascular disease and a severely reduced EF of 5%. Yet, a combination of GDMT and surgical valve repair extended survival and improved quality of life. The use of beta-blockade therapy remains the standard of care for the medical management of MFS, regardless of aortic dilation. A major goal in treatment also includes adequate blood pressure control with the addition of an angiotensin receptor blocker. The standard treatment of valvular disease, such as mitral valve prolapse and mitral and aortic valve regurgitation, is surgical repair. This patient’s unique clinical trajectory - marked by progressive valvular degeneration, reduced cardiac output, and limited access to healthcare - emphasizes the critical need for continuity of care, even in late-stage disease. He was started on metoprolol for beta-blockade and the sacubitril/valsartan combination therapy for decompensating heart failure, in addition to other home medications like spironolactone and furosemide for blood pressure control. Beta-blockers are recommended primarily for aortic dilation to reduce stress on the aorta. If no dilation is present, their role is less definitive unless other indications exist (e.g., arrhythmia or heart failure). Angiotensin II receptor blockers (e.g., losartan) have been studied as potential alternatives or adjuncts to beta-blockers in reducing aortic dilation progression. However, in this case, sacubitril/valsartan was used primarily for heart failure, not specifically for MFS-related vascular protection. The placement of an AICD was pursued as a preventive measure against sudden cardiac death, which is a known risk in MFS patients with significantly reduced EF, although studies remain limited on long-term outcomes in this specific subset.

Valve repair is preferred over replacement when feasible to preserve native valve function, particularly for mitral valve prolapse. Also, aortic valve intervention in MFS often involves aortic root replacement rather than isolated valve replacement. The prognosis of MFS has improved dramatically with modern surgical and medical therapies. Social limitations are important, but specific challenges should be mentioned (e.g., financial barriers, transportation issues, and lack of social support). Social limitations included financial instability, inconsistent transportation to follow-ups, and lack of support, all of which affected medication adherence and timely care. The involvement of social work and case management is crucial in coordinating access to telehealth, pharmacy delivery, and cardiology follow-up, contributing to better continuity of care. In general, beta-blockade remains the cornerstone of MFS management regardless of aortic dilation, with angiotensin receptor blockers playing an increasing role in limiting aortic growth and reducing vascular wall stress. Surgical repair continues to be the mainstay for severe mitral and aortic regurgitation. While this aligns with standard MFS care, the delayed recognition of physical features, social barriers to follow-up, and the need for advanced interventions make this case particularly compelling. The life expectancy of individuals with MFS can be near normal with appropriate management. However, once chronic cardiovascular disease sets in, particularly in the form of heart failure or aortic dissection, life expectancy decreases by nearly one-third. This patient’s late diagnosis placed him at significant risk, but his consistent follow-up, surgical intervention, and adherence to medical therapy helped stabilize his course. Moving forward, his prognosis will depend on continued blood pressure management, surveillance of aortic dimensions, and regular cardiology follow-up. Despite the challenges, this case reinforces the idea that even delayed intervention in MFS can yield meaningful improvement in outcomes when guided by a comprehensive, multidisciplinary approach.
